# Should personalised dosing have a role in cancer treatment?

**DOI:** 10.3389/fonc.2023.1154493

**Published:** 2023-05-05

**Authors:** Claire C. Villette, David Orrell, Jim Millen, Christophe Chassagnole

**Affiliations:** Physiomics PLC, Oxford, United Kingdom

**Keywords:** cancer treatment, G-CSF, DOCETAXEL, prostate cancer, dose optimization, PK/PD model, neutropenia, personalised dosing

## Abstract

Almost all pharmaceutical products are approved on the basis of their effect in patients representing the “average” of the population studied in registrational trials, with most drug labels allowing, at most, for empirical dose reduction in the case of toxicity. In this perspective article we explore some of the evidence that supports the use of personalised dosing in cancer treatment and show how we have been able to build on existing models linking dose, exposure and toxicity to demonstrate how dose optimisation, including increasing the dose, has the potential to significantly improve efficacy outcomes. We also explore, through the lens of our own experience of developing a personalised dosing platform, some of the hurdles that stand in the way of implementing a personalised approach to dosing in real world settings. In particular, our experience is illustrated by the application of a dosing platform for docetaxel treatment in prostate cancer.

## 1 Introduction

Drug development is a notoriously expensive and risky business. It is estimated that it costs around a billion dollars, from initial design to final patient trials, to develop a successful new treatment (1). The vast majority of new candidates of course do not get this far. And even if a drug does make it to market, the actual improvement that it offers over existing treatments may not be very great. For example, one study found that between 2008 and 2012 the FDA approved most uses of cancer drugs based on surrogate outcomes, such as response rate or progression-free survival, with limited or no evidence of overall survival or improved quality of life. Where measured, median improvement in survival among cancer patients treated with 71 different anti-cancer drugs was just 2.1 months (2). Real world benefits are often significantly less than those measured in trials.

While the quest for new drugs is of course essential, an alternative and much less expensive approach to improving outcomes is to use existing drugs more efficiently, by concentrating on questions such as dosage and timing. There is increasing evidence that drugs can have a very different effect depending on the details of how and when they are taken, and on who they are taken by; and unlike the design of a radical new compound, such questions are often amenable to analysis using mathematical models. It is much easier to test and optimise a treatment protocol in silico for an existing drug, whose properties and mechanism of action are well-understood, than it is to predict the effect of a new drug.

This perspective paper will look at how mathematical models can be used to optimise anti-cancer treatments on a personalised basis, and discuss some barriers that still hinder their clinical use. A range of examples will be highlighted, including a focus on a specific precision dosing application for prostate cancer treatment that will serve as in-depth illustration.

## 2 Dose optimisation using mathematical models

One example of the use of mathematical models to optimise treatment, is in the scheduling for anti-cancer drugs. These drugs are often used in combination, and the interaction between them will typically depend on complex, time-dependent effects; for example, one treatment might directly inhibit or potentiate another treatment, or create synchronisation effects in the cell population, which mean that the effect of treatment is highly time-sensitive. This fact has attracted the attention of clinicians who are looking at increasingly complex schedules in order to obtain substantial gains in survival rates (3). Mathematical models of growing tumours have been developed which allow modellers to predict the effect of a change in schedule and optimise treatment (4).

Another type of schedule-dependent effect is the sort seen with chronotherapy, where the impact of a drug varies substantially depending on the time of day at which it is taken, or more precisely the patient’s circadian cycle. For example, results from three phase three trials in metastatic colorectal cancer suggest a superiority of chronotherapeutic vs conventional schedules in man with a gain in overall survival, 20.8 months vs 17.5 months, whereas a reverse effect was observed in females (5). Rather than develop a new drug from scratch, a similar gain in efficacy can therefore be obtained by using the existing drug more optimally, in a manner which may depend on the patient (since circadian cycles vary). Again, mathematical models have been developed to simulate and predict chronotherapeutic schedules (6, 7).

These types of schedule optimization focus on the timing of drug interventions, but equally important and getting increasing attention (8, 9) is the response of the individual patient, in terms of both the compound’s efficacy, and its toxicity. Suppose for example that one patient has unusually high tolerance for a drug, so experiences little in the way of toxicity. Then it may be appropriate to give a higher, and possibly more efficacious dose, to that patient, as compared to another patient who has trouble tolerating the standard dose.

While in principle it might be possible to predict such effects for certain compounds based on things like genetic information, a more direct approach is to monitor the patient’s response to the dose, using PK or proxy biomarker measurements, and predict from that whether (and to what level) the dose should be increased or lowered. For instance, PK-guided dosing of 5-Fu in metastatic colorectal cancer led to an increase in median overall survival from 16 to 22 months (8). Similar techniques applied to docetaxel dosing in a feasibility study were able to reduce the inter-individual variability in exposure and white blood cell count decrease by 39% and 50%, respectively (10). A further study in a larger population would be needed to clarify the clinical outcomes in terms of efficacy and toxicity.

Such precision-dosing techniques typically require costly additional tests which severely restrict their use in clinical practice. In the sections below, however, we describe a software application (or app) which uses a mathematical model to generate personalized dose recommendations for treatment of prostate cancer, and which requires only a weekly standard blood test in the first chemotherapy cycle. This app aims to significantly improve patient outcome at a low cost without disrupting the current clinical practice treatment pathway.

## 3 A precision dosing application for docetaxel in prostate cancer

Among males, prostate cancer is the most common cancer in Europe and the second most common cancer worldwide, especially in those aged above 70 years. Each year there are typically around 50,000 diagnoses of prostate cancer in the UK, and 170,000 in the USA. The main chemotherapy treatment for advanced-stage disease is three-week cycles of Docetaxel. A common toxic side-effect of this treatment is neutropenia, which must be controlled because it leads to a heightened risk of severe infection. The appropriate dose is therefore determined from population studies, with an individual’s dose normally scaled by their body surface area. Granulocyte colony-stimulating factor (G-CSF) is also often used to combat neutropenia.

While treatment is aimed at the “average” patient, inter-individual variability in docetaxel exposure (area under the concentration profile curve) has been observed to cover a 2-3-fold range for the same administered dose level (11). Furthermore, the toxicity of Docetaxel also varies from patient to patient, and since the dose is standardised, this means that an estimated 20 percent of patients may be overdosed, and another 30 percent of patients may be underdosed (10, 12). In other words, the standard treatment is only well-matched to about half the patient population.

An app was developed which uses a mathematical model to generate personalized dose recommendations for treatment of prostate cancer in order to better serve the other half, by tuning the dose so that the treatment gives the optimal balance between efficacy and toxicity (13–15). The main feature of the dosing app is to use neutrophil count as a proxy biomarker for docetaxel exposure.

A population pharmacokinetics (PK) model for docetaxel (10), and a pharmacodynamic (PD) model for myelosuppression are first combined in order to produce a patient-specific PK/PD toxicity model.

The population PD model relates docetaxel concentration (exposure) to neutrophil count using a system of differential equations. It is based on well-established chemotherapy-induced myelosuppression models (16–18), adapted to both endogenous (naturally produced) and exogenous (administered as co-medication) G-CSF effects, in particular the simulation of proliferation and maturation of progenitor cells.

The combined PK/PD model has five patient-specific parameters (PK elimination rate, baseline neutrophil count, sensitivity of hematopoietic cells to docetaxel, hematopoietic cells proliferative feedback, and maturation time) publicly available data from the comparator arm of a phase III clinical trial for metastatic hormone-resistant prostate cancer (clinical trial number NCT00617669, see (19)). The model was also tested to capture the mean neutrophil profile from two arms of a clinical trial (20) comparing chemotherapy alone and chemotherapy plus filgrastim.

Finally, proportional hazards models (Cox models) for overall survival and progression-free survival were developed (13) which relate Docetaxel exposure and patient baseline biomarker levels such as LDH, ALP and PSA to survival time probability, and allow estimation of the gains in prognosis that can be achieved by modifying the patient dose after the first chemotherapy cycle. A similar approach has been reported which relates docetaxel myelosuppression effect to survival in non-small cell lung cancer patients (21).

In the current standard of care, the first docetaxel dose is selected based on patient BSA. A blood sample is routinely collected on the day of first injection and sometimes on the first day of each subsequent cycle. If clinical toxicity is observed, the next dose is reduced and/or accompanied by G-CSF, or the treatment is terminated. Docetaxel is administered in 3-week cycles until switch to another line of treatment, unacceptable toxicity or death.

This precision dosing app requires two additional blood tests during the first chemotherapy cycle around day 7 and day 14. Just before selecting the second chemotherapy dose, patient characteristics (height, weight) and blood tests results are entered into the app to compute patient-specific model parameters. These models then output expected patient neutropenia levels and prognosis compared to the general patient population. Simulations of dose changes can be run in seconds.

**Figure 1:**
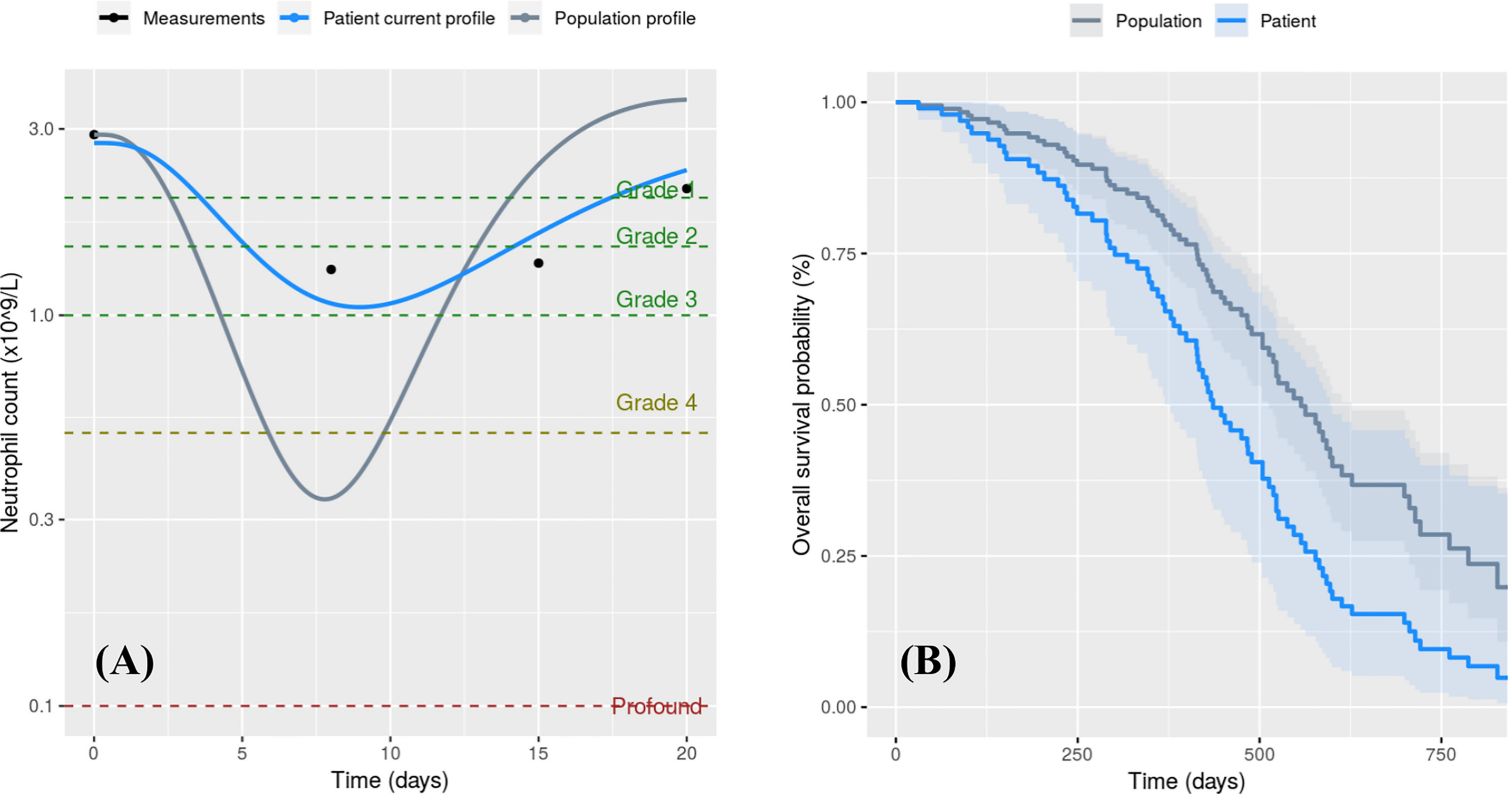
Patient-specific (blue) and population median (grey) simulations associated with a 75mg docetaxel course. **(A)** Neutrophil counts over the first cycle. Clinical measurements for this particular patient are shown as black dots and neutropenia grades are depicted by dashed lines. **(B)** Overall survival probability.

The combined predictions of the toxicity and survival models on the calibration dataset suggested some patients might have benefitted from a dose reduction (limiting toxicity while maintaining relatively good survival chance) or a dose increase (**Figure 1**) (increasing survival chance while maintaining neutropenia at a manageable level). Such simulations ran as part of the treatment pathway may help clinicians to identify effective, under or overdosing and modulate the docetaxel dose and G-CSF co-medication accordingly.

## 4 Barriers to model-based dosing optimisation in practice

Despite the clear evidence in favour of dosing optimisation there are many practical considerations related to cost, clinical guidelines, patient convenience, physician preference and adoption of new technologies. We will explore some of these in this section.

Depending on the standard of care for the specific indication and the frequency with which patients attend for blood tests, additional patient visits may be required to take blood samples and generate additional neutrophil counts required to calibrate the model. This has a cost and time implication for the patient and the health system and may require formal amendments to care pathways, which although justified still represent an additional hurdle to overcome.

When treating some cancers other than prostate using myelosuppressive drugs, patients may routinely be monitored more frequently and there could be value in exploring the use of model based dosing for these (for example chemo-radiotherapy and some haematological cancers). Another possible solution to the issue of higher sampling frequencies is the use of point-of-care devices under development that allow clinicians or even patients to obtain cell counts based on small volumes of blood using technologies that are compact and suitable for use in outpatients, primary care or even a patient’s own home. Results from such devices could then be uploaded to a hospital’s electronic medical record system (possibly *via* a phone-based app) and viewed alongside other blood results taken by more traditional routes.

Finally, the benefits that can accrue from dosing optimisation rely on the active participation of clinicians and other involved healthcare personals to both facilitate the collection of the data required for model calibration (for example by authorising additional visits/tests) and perhaps more importantly on their willingness to engage with the model and weigh the information it presents to them alongside other data including their own personal experience, all in the context of each individual patient’s situation. An example of such interactions have been described by Barnett et al. (9), showing how it can benefit childhood cancer patient, e.g. by reducing the risk of relapse (22), whilst it can be still challenging to implement. One of the logistic limitations being the measurement of drug concentrations, that could potentially be overcome by more easily measurable proxy biomarker such as neutrophil.

## 5 Discussion

In this paper we have argued for the use of mathematical models in optimising doses and schedules, and presented an app for personalised treatment of prostate cancer. An advantage of the modelling approach is that it offers a framework for incorporating and combining information, which can be continuously updated as relevant data becomes available. For the prostate cancer app, the dose is adjusted in response to blood tests which reveal the level of neutropenia.

While such models offer many advantages to clinicians, their use also involves a change in working practices. Instead of having a single approved maximum dose, with set rules for modifying it, the optimal dose may need to be increased or decreased. This information has to be conveyed to other parties, such as the pharmacist. Also, doctors may be more confident in their own ability to prescribe treatments, as opposed to consulting an app. This means that, in order to be widely adopted, the model-based approach to personalised dosing has to provide a very clear level of benefit over existing methods. The challenge is great, but so is the potential for improving therapies and clinical outcomes for patients.

## Data availability statement

Publicly available datasets were analyzed in this study. This data can be found here: Clinical trial number NCT00617669 https://data.projectdatasphere.org/projectdatasphere/html/home.

## Author contributions

All authors participated in discussions regarding the concept/design and topics reviewed in this manuscript. CV performed the model simulations and data analyses presented in Section 3. The manuscript was written by CV and DO, and reviewed by CC and JM. All authors contributed to the article and approved the submitted version.

## References

[1] Wouters OJ McKee M Luyten J. Estimated research and development investment needed to bring a new medicine to market, 2009-2018. JAMA (2020) 323(9):844. 32125404 10.1001/jama.2020.1166 PMC7054832

[2] Prasad V. Do cancer drugs improve survival or quality of life? BMJ (2017), j4528. 28978548 10.1136/bmj.j4528 PMC5695531

[3] Raben D Bunn PA. Biologically targeted therapies plus chemotherapy plus radiotherapy in stage III non-Small-Cell lung cancer: a case of the Icarus syndrome? J Clin Oncol (2012) 30(32):3909–12. 10.1200/JCO.2012.43.1866 23045597

[4] Orrell D Fernandez E. Using predictive mathematical models to optimise the scheduling of anti-cancer drugs. Innov Pharm Technol (2010), 60–2.

[5] Giacchetti S Perpoint B Zidani R Le Bail N Faggiuolo R Focan C. Phase III multicenter randomized trial of oxaliplatin added to chronomodulated fluorouracil-leucovorin as first-line treatment of metastatic colorectal cancer. J Clin Oncol (2000) 18:136–47. 10.1200/JCO.2000.18.1.136 10623704

[6] Hesse J Martinelli J Aboumanify O Ballesta A Relógio A. A mathematical model of the circadian clock and drug pharmacology to optimize irinotecan administration timing in colorectal cancer. Comput Struct Biotechnol J (2021) 19:5170–83. doi: 10.1016/j.csbj.2021.08.051 PMC8477139 34630937

[7] Ballesta A Innominato PF Dallmann R Rand DA Lévi FA. Systems chronotherapeutics. Pharmacol Rev (2017) 69(2):161–99. doi: 10.1124/pr.116.013441 PMC5394920 28351863

[8] Gamelin E Delva R Jacob J Merrouche Y Raoul JL Pezet D. Individual fluorouracil dose adjustment based on pharmacokinetic follow-up compared with conventional dosage: results of a multicenter randomized trial of patients with metastatic colorectal cancer. J Clin Oncol (2008) 26(13):2099–105. doi: 10.1200/JCO.2007.13.3934 18445839

[9] Barnett S Holden V Campbell-Hewson Q Veal GJ. Perspectives and expertise in establishing a therapeutic drug monitoring programme for challenging childhood cancer patient populations. Front Oncol (2022) 11:815040. doi: 10.3389/fonc.2021.815040 35071019 PMC8770741

[10] Engels FK Loos WJ van der Bol JM de Bruijn P Mathijssen RHJ Verweij J. Therapeutic drug monitoring for the individualization of docetaxel dosing: a randomized pharmacokinetic study. Clin Cancer Res (2011) 17(2):353–62. doi: 10.1158/1078-0432.CCR-10-1636 21224369

[11] McLeod HL Kearns CM Kuhn JG Bruno R. Evaluation of the linearity of docetaxel pharmacokinetics. Cancer Chemother Pharmacol (1998) 42:155–9. doi: 10.1007/s002800050799 9654116

[12] Gurney H. How to calculate the dose of chemotherapy. Br J Cancer (2002) 86(8):1297–302. doi: 10.1038/sj.bjc.6600139 PMC2375356 11953888

[13] Villette C Mistry H Ortega F Orell D Brightman F Millen J. A precision dosing application for advanced prostate cancer chemotherapy. Cancer Res (2019) 79(13 Suppl):67.

[14] Hurry C Villette C Mistry H Millen J Chassagnole C. Abstract 228: a precision dosing application for patients treated with docetaxel and G-CSF. Cancer Res (2021) 81(13_Supplement):228–8. doi: 10.1158/1538-7445.AM2021-228

[15] Villette C Hurry C Mistry H Millen J Chassagnole C. A precision dosing application for prostate cancer patients treated with docetaxel and G-CSF. JCO 39(15_suppl):e13585–5. doi: 10.1200/JCO.2021.39.15_suppl.e13585

[16] Friberg LE Henningsson A Maas H Nguyen L Karlsson MO. Model of chemotherapy-induced myelosuppression with parameter consistency across drugs. J Clin Oncol (2002) 20(24):4713–21. doi: 10.1200/JCO.2002.02.140 12488418

[17] Netterberg I Nielsen EI Friberg LE Karlsson MO. Model-based prediction of myelosuppression and recovery based on frequent neutrophil monitoring. Cancer Chemotherapy Pharmacol (2017) 80(2):343–53. doi: 10.1007/s00280-017-3366-x PMC5532422 28656382

[18] Quartino AL Friberg LE Karlsson MO. A simultaneous analysis of the time-course of leukocytes and neutrophils following docetaxel administration using a semi-mechanistic myelosuppression model. Invest New Drugs (2012) 30(2):833–45. doi: 10.1007/s10637-010-9603-3 21153753

[19] Fizazi K Higano CS Nelson JB Gleave M Miller K Morris T. Randomized, placebo-controlled study of docetaxel in combination with zibotentan in patients with metastatic castration-resistant prostate cancer. J Clin Oncol (2013) 31(14):1740–7. doi: 10.1200/JCO.2012.46.4149 23569308

[20] Crawford J Ozer H Stoller R Johnson D Lyman G Tabbara I. Reduction by granulocyte colony-stimulating factor of fever and neutropenia induced by chemotherapy in patients with small-cell lung cancer. N Engl J Med (1991) 325(3):164–70. doi: 10.1056/NEJM199107183250305 1711156

[21] Lombard A Mistry H Aarons L Ogungbenro K. Dose individualisation in oncology using chemotherapy-induced neutropenia: example of docetaxel in non-small cell lung cancer patients. Br J Clin Pharmacol (2020), 14614. 10.1111/bcp.14614 33075149

[22] Evans WE Relling MV Rodman JH Crom WR Boyett JM Pui CH. Conventional compared with individualized chemotherapy for childhood acute lymphoblastic leukemia. N Engl J Med (1998) 338(8):499–505. doi: 10.1056/NEJM199802193380803 9468466

